# Frequency and Associated Factors of Interruptions During the Medication Administration Process Among Nurses in South Korea: A Cross‐Sectional Study

**DOI:** 10.1111/jan.70321

**Published:** 2025-10-28

**Authors:** Seung Gyeong Jang, Eun Young Choi, Seung Ju Baek, Hye‐Mi Moon, Sang Hee Hong, Jin Kyung Cho, Won Lee

**Affiliations:** ^1^ College of Nursing Inje University Gimhae Republic of Korea; ^2^ Department of Nursing Chung‐Ang University Seoul Republic of Korea; ^3^ Department of Nursing, the Graduate School Chung‐Ang University Seoul Republic of Korea; ^4^ Nursing Division Chung‐Ang University Gwangmyeong Hospital Gwangmyeong‐si Republic of Korea; ^5^ Nursing Division Chung‐Ang University Hospital Seoul Republic of Korea

**Keywords:** cultural issues, medication, nurses, patient safety, work organisation, workflow interruptions

## Abstract

**Aim (s):**

To investigate the frequency and associated factors of interruptions initiated by human and environmental sources during the medication administration process among nurses in South Korea.

**Design:**

A cross‐sectional descriptive study.

**Methods:**

Data were collected from January to March 2022 through an online survey administered to nurses working in tertiary hospitals in South Korea. The survey assessed interruptions during the medication administration process, nursing work environments and organisational culture. Descriptive statistics and regression analysis were used to identify factors associated with interruptions.

**Results:**

Human‐initiated interruptions were more frequent than those initiated by environmental sources. Human‐initiated interruptions increased with a higher patient load and a relation‐oriented organisational culture but decreased with adequate staffing and resources, as well as an innovation‐oriented culture. Environment‐initiated interruptions were more frequent in settings with a task‐oriented culture and less frequent among female nurses.

**Conclusion:**

The findings highlight the importance of understanding the distinct characteristics of interruptions and developing targeted strategies based on their sources and contributing factors. Creating supportive environments and fostering an organisational culture that actively prevents unnecessary interruptions are essential for enhancing medication safety and workflow efficiency.

**Implications for the Profession and/or Patient Care:**

To apply these findings in clinical practice, it is necessary to allocate staffing resources appropriately to reduce interruptions. Providing education on the importance of maintaining uninterrupted medication administration processes is essential to reduce human‐initiated interruptions.

**Impact:**

This study provides practical evidence that organisational culture and staffing are associated with interruptions in clinical nursing practice. Nurse managers should apply these findings by promoting staffing adequacy and fostering a collaborative, innovative environment that encourages continuous improvement and openness to change. Tailored strategies that reflect the specific characteristics of different types of interruptions can help reduce their occurrence and improve medication safety.

**Reporting Method:**

STROBE checklist.

**Patient or Public Contribution:**

No patient or public contribution.

**Summary**

- What does this paper contribute to the wider global clinical community?
  ○ This study contributes valuable knowledge on the factors associated with interruptions during the medication administration process in nursing, particularly within the context of organisational culture and the work environment.
  ○ These findings highlight the importance of interventions tailored to organisational and contextual factors to reduce interruptions and enhance nursing practice across diverse healthcare systems.
  ○ The study's emphasis on organisational culture and environmental factors provides a broader understanding that can be applied across diverse healthcare settings, ultimately contributing to safer patient care and better nursing outcomes worldwide.

## 1 Introduction

The medication administration process (MAP) refers to medication‐related clinical tasks performed by nurses, including prescription verification, preparation, administration and other related activities (Johnson et al. 2017; Schroers 2018). Medication is one of the most critical nursing tasks, constituting a substantial portion of nurses' clinical workload, and nurses play a central role in ensuring its safety (Kim et al. 2023). Despite the high stakes associated with this responsibility, nurses frequently experience various work interruptions during the MAP (Eid et al. 2022; Wang et al. 2021).

Work interruptions can primarily lead to medication errors, which are a leading cause of preventable patient harm and continue to occur at high rates globally despite various efforts to reduce them (Johnson et al. 2017; Webster 2022). Additional negative consequences, such as decreased concentration, emotional exhaustion, stress‐related symptoms and a sense of frustration among nurses, have also been associated with work interruption (Eid et al. 2022; Johnson et al. 2017; Laustsen and Brahe 2018). Such consequences underscore the urgent need to address interruptions during the MAP from both a patient safety and nursing quality perspective (Johnson et al. 2017).

Given the direct impact of work interruptions on patient safety, several studies have investigated the frequency, sources, and outcomes of work interruptions during medication administration (Schroers 2018). However, many of these studies have relied on observational methods, such as direct observation, which provide objective and specific data but have limitations in comprehensively understanding the phenomenon (Danesh et al. 2022). More importantly, although organisational factors such as work environment and organisational culture play a critical role in shaping how nurses experience and respond to work interruptions (Hopkinson and Wiegand 2017; Laustsen and Brahe 2018), few studies have systematically examined these organisational aspects. Since organisational factors are closely related to nurses' workflow and responses to work interruptions, a more comprehensive understanding is required to inform context‐specific interventions (Biron et al. 2009; Jalali et al. 2025).

The Korean healthcare system exemplifies the importance of examining organisational factors within unique cultural contexts. Key features include the presence of family caregivers at patients' bedsides, higher nurse‐to‐patient ratios compared to Western healthcare systems, and a hierarchically structured nursing culture (Kim and Oh 2016; Park et al. 2022; Hong et al. 2023). Some of these organisational and cultural characteristics are not unique to Korea but represent common patterns across Asian healthcare systems, making research findings from Korea valuable for understanding similar contexts globally (Park et al. 2022). Therefore, investigating organisational factors associated with work interruptions in the Korean healthcare context can provide insights that are applicable to similar healthcare systems worldwide and contribute to the development of culturally sensitive interventions for increasingly diverse healthcare environments.

## 2 Background

### 2.1 Work Interruptions During the MAP

Work interruptions during the MAP are of particular concern, as studies have shown that these interruptions account for a substantial proportion of nurses' work interruptions during clinical shifts (Kellogg et al. 2021; Kwon et al. 2021). Interruptions during the MAP are especially problematic because prolonged and frequent interruptions can distract the nurses' attention and directly contribute to medication errors (Bower et al. 2015).

Observational studies have identified various sources of interruptions during the MAP, including nurses, physicians, patients, caregivers, phone calls, equipment alarms or failures, and missing supplies (Eid et al. 2022; Johnson et al. 2017). However, these studies have found varying patterns in interruption frequency, characteristics and impacts (Eid et al. 2022; Johnson et al. 2017). These discrepancies may be attributed to differences in clinical unit characteristics, institutional context, and measurement methods employed (McGillis Hall et al. 2010). Measuring work interruptions and understanding nurses' perspectives on their sources within clinical contexts are essential for developing targeted strategies to reduce work interruptions (Lin et al. 2021; Yu and Lee 2022; Wang et al. 2024).

### 2.2 Theoretical Framework: The SEIPS Model

To understand the multiple factors related to work interruptions, this study adopts the Systems Engineering Initiative for Patient Safety (SEIPS) model as its theoretical framework for examining work interruptions (Holden et al. 2013). The SEIPS model emphasises how components of a work system interact to shape healthcare processes and outcomes (Holden et al. 2013). The model is grounded in the principle that healthcare outcomes result from the complex interplay between multiple work system elements rather than isolated factors. The model presents six key components: person, task, tools and technologies, physical environment, organisation and external environment. This study primarily focuses on the organisational component of the SEIPS framework while incorporating person (nurses' individual characteristics), task (nurse‐to‐patient ratios) and physical environment (ward type) factors as key variables.

### 2.3 Organisational Culture and Work Interruption During the MAP

Organisational culture represents a fundamental organisational component that significantly shapes how nurses experience and interpret work interruptions. This cultural dimension forms part of the organisation's broader social context and influences staff behaviour and perceptions (Trus et al. 2019). In certain cultural climates, work interruptions are normalised and embedded within the clinical workflow (Yu et al. 2019). Nurses often perceive work interruptions as an inevitable part of their routine, with decisions to interrupt others based on the urgency and significance of the task (Laustsen and Brahe 2018; Wang et al. 2024). Additionally, nurses' attitudes toward work interruptions during the MAP are significantly influenced by previous experience and nursing culture (Wang et al. 2024). These findings underscore the importance of exploring how nursing organisational culture is associated with the perception of work interruptions during the MAP.

### 2.4 Work Environment and Work Interruption During the MAP

The work environment constitutes another essential organisational component that directly affects the occurrence of work interruptions. Structural challenges, such as increasing workloads and rapidly changing clinical environments, have been reported to influence work interruptions by making it difficult for nurses to maintain a consistent workflow (Son and Kim 2019). Adequate staffing and resource allocation enable nurses to focus on medication tasks without frequent disruptions (Liu et al. 2025). In contrast, staff shortages and excessive workloads impair nurses' ability to manage interruptions effectively, increasing their burden and heightening the risk of medication errors. Notably, work interruptions can be reduced through organisational coordination and work environment improvements, suggesting that work interruptions are not merely individual issues but rather organisational challenges that can be mitigated through systematic interventions (McGillis Hall et al. 2010).

Measuring work interruptions and understanding nurses' perspectives on their sources within clinical contexts are essential for developing targeted strategies to reduce work interruptions (Yu and Lee 2022; Wang et al. 2024). Particularly, empirical investigation into the impact of organisational culture and the work environment on work interruptions during the MAP within the aforementioned unique cultural and organisational context of Korea is needed.

## 3 The Study

This study aimed to investigate the frequency and associated factors of work interruptions during the MAP among nurses in South Korean hospitals.

## 4 Methods

### 4.1 Study Design

This study employed a cross‐sectional design using online survey questionnaires at tertiary hospitals in South Korea.

### 4.2 Participants

The inclusion criteria for the study participants were as follows: (1) nurses who could perform nursing tasks independently within their department; (2) nurses who had been working continuously in their current department for at least 6 months; (3) nurses who performed medication tasks (including all processes from medication preparation to direct administration to patients) during their working hours. The exclusion criteria were as follows: (1) nurses working in outpatient clinics, operating rooms or recovery rooms and (2) nurse managers at the level of head nurse or higher. Participants were recruited using a combination of convenience and snowball sampling methods, based on accessibility and voluntary participation across the hospitals. In four tertiary hospitals that formally agreed to participate, survey links were posted on the hospitals' official intranet bulletin boards after obtaining approval from the nursing departments. When additional participant recruitment was needed, snowball sampling was used through nurse community communication channels (e.g., group chat rooms) in three other hospitals.

We intended to use multiple regression analysis to identify the key factors associated with work interruptions during MAP. Therefore, the required sample size was calculated using the G‐power 3.1.9.7 program (Faul et al. 2007). The minimum sample size was determined to be 204 based on a 0.15 median effect size (f), a significance level of 0.05, a power of 0.95 and 16 predictors. Considering potential dropouts, data were collected from 263 nurses who completed the survey.

### 4.3 Measurements

A summary of the instruments, including domains, items and response scales, is presented in Table S1. Given the study's focus and the characteristics of the instruments, analyses were conducted using domain‐level scores, and Cronbach's α was reported at the domain level accordingly.

#### 4.3.1 Frequency of Work Interruption During the MAP

In this study, work interruptions during the MAP are defined as breaks in the continuity of medication tasks due to various factors. The frequency of work interruption during the MAP was measured using the Nursing Work Interruption Scale (Yu and Lee 2022), with the term ‘work’ specifically modified to ‘the medication administration process’ for this study. This scale comprised 12 items across two domains: human‐initiated (six items) and environment‐initiated (six items) interruptions. Responses were measured on a 6‐point Likert scale ranging from ‘almost none’ (1 point) to ‘at least 5 times per day’ (6 points). Higher total scores indicated a higher frequency of perceived interruptions during the MAP. At the time of the scale's development, Cronbach's α for the domains ranged from 0.83 to 0.84 (Yu and Lee 2022). In this study, Cronbach's α for the domains ranged from 0.81 to 0.92.

#### 4.3.2 Nursing Work Environments

Nursing work environments were measured using the Korean version of the Practice Environment Scale of Nursing Work Index (K‐PES‐NWI) originally developed by Lake (2002) and translated into Korean by Cho et al. (2011). The original tool comprised 29 items across five domains: ‘nurse participation in hospital affairs’ (nine items); ‘nursing foundations for quality of care’ (nine items); ‘nurse managers' ability, leadership and support of nurses’ (four items); ‘staffing and resource adequacy’ (four items); ‘collegial nurse–physician relations’ (three items). For this study, four domains, excluding ‘nurse participation in hospital affairs’, were used. Responses were measured on a 4‐point Likert scale ranging from ‘strongly disagree’ (1 point) to ‘strongly agree’ (4 points). Higher total scores indicated better nursing work environments. At the time of Korean version development, Cronbach's α for the domains ranged from 0.80 to 0.84 (Cho et al. 2011). In this study, Cronbach's *α* for domains ranged from 0.74 to 0.86.

#### 4.3.3 Nursing Organisational Culture

Nursing organisational culture was measured using the Nursing Organisational Culture Measurement Tool developed by Kim et al. (2004). This tool comprised 20 items across four cultural domains: ‘relation‐oriented culture’ (five items), ‘innovation‐oriented culture’ (six items), ‘hierarchy‐oriented culture’ (five items) and ‘task‐oriented culture’ (four items). Responses were measured on a 5‐point Likert scale ranging from ‘strongly disagree’ (1 point) to ‘strongly agree’ (5 points). Higher scores in each cultural domain indicated a stronger perception of that particular culture type. At the time of development, Cronbach's α for the domains ranged from 0.71 to 0.87 (Kim et al. 2004). In this study, Cronbach's α for domains ranged from 0.67 to 0.91.

#### 4.3.4 General Characteristics

The general characteristics of the participants included individual characteristics (gender, age, work experiences as a nurse), work‐related factors (average number of patients per nurse per shift), and workplace characteristics (type of ward).

### 4.4 Data Collection

Data were collected from January to March 2022 through an online survey. The online link included a feature restricting multiple entries from the same user, ensuring anonymity and voluntary participation. The survey took approximately 20 min, and the participants received a mobile coupon worth 10,000 KRW (approximately 8 USD) upon completion.

### 4.5 Data Analysis

Data were analysed using SPSS version 25.0 (IBM Corp., Armonk, NY, USA). All responses were complete, and no missing data was identified in the dataset. Frequencies, percentages, means, and standard deviations were calculated for each variable. Further, t‐tests or one‐way analysis of variance (ANOVA) were conducted to examine the differences in the frequency of work interruptions according to the general characteristics of the nurses. Multiple linear regression analysis was performed to examine the associations between the nursing work environment, nursing organisational culture, and the frequency of work interruptions during the MAP among nurses.

### 4.6 Ethical Considerations

This study was approved by the Institutional Review Board of Chung‐Ang University (Approval Number: 1041078‐202107‐HRSB‐221). The survey was conducted anonymously, and nurses who wished to participate voluntarily after reviewing the research announcement and explanation joined the survey. During data collection, personal information was limited to the minimum necessary for the study, such as age, gender, work experience, and educational level, as well as information for compensation (phone number), and participants' anonymity was ensured.

## 5 Results

### 5.1 Participants' Characteristics

Most of the participants were female (95.4%), the average age was 30.43 ± 5.01 years old, and the average work experience as a nurse was 7.53 ± 5.28 years. In total, 79.8% of nurses worked in a general ward; the average number of patients per nurse per shift was 8.94 ± 4.25 (Table 1).

**TABLE 1:** General characteristics of participants and frequency of work interruptions during the MAP (*N* = 263).

Category	*n* (%) or Mean ± SD		Frequency of work interruption during the MAP			
			Human‐initiated		Environment‐initiated	
			Mean ± SD	*t*/*F* (*p*)	Mean ± SD	*t*/*F* (*p*)
Gender						
Male	12	(4.6)	15.41 ± 6.37	−0.34 (0.73)	13.50 ± 4.30	1.75 (0.08)
Female	251	(95.4)	16.19 ± 7.72		11.06 ± 4.75	
Age	30.43 ± 5.01					
20–29 years	130	(49.4)	15.45 ± 7.18	1.46 (0.23)	10.64 ± 4.62	1.73 (0.18)
30–39 years	115	(43.7)	17.07 ± 8.29		11.77 ± 4.95	
≥ 40 years	18	(6.8)	15.44 ± 6.46		11.22 ± 4.10	
Work experience as a nurse	7.53 ± 5.28					
≤ 5 years	100	(38.0)	15.59 ± 6.95	0.73 (0.49)	10.42 ± 4.34	2.06 (0.13)
5–10 years	92	(35.0)	16.90 ± 8.32		11.57 ± 4.82	
> 10 years	71	(27.0)	15.98 ± 7.76		11.72 ± 5.12	
Type of ward						
General ward	210	(79.8)	16.65 ± 7.69	2.24 (0.11)	11.06 ± 4.79	1.66 (0.19)
Intensive care unit	43	(16.3)	14.07 ± 6.95		12.14 ± 4.79	
Emergency room	10	(3.8)	14.70 ± 8.92		9.40 ± 2.91	
Average number of patients per nurse per shift	8.94 ± 4.25					
Below the median	120	(45.6)	14.23 ± 7.30	−3.84 (< 0.001)	10.73 ± 4.80	−1.37 (0.17)
Above the median	143	(54.4)	17.78 ± 7.60		11.54 ± 4.71	

Abbreviations: MAP, medication administration process; SD, standard deviation.

In the nursing work environment, ‘nursing foundations for quality of care’ had the highest score (an average of 2.57 ± 0.44 points), while ‘staffing and resource adequacy’ had the lowest score (an average of 1.83 ± 0.59 points). In nursing organisational culture, ‘hierarchy‐oriented culture’ had the highest score (an average of 3.67 ± 0.63 points), while ‘innovation‐oriented culture’ had the lowest score (an average of 2.79 ± 0.80 points) (Table 2).

**TABLE 2:** Scores of nursing work environments and nursing organisational culture (*N* = 263).

Variables	Mean ± SD
**Nursing work environments**	
Nursing foundations for quality of care	2.57 ± 0.44
Nursing managers' ability, leadership and support of nurses	2.51 ± 0.60
Staffing and resource adequacy	1.83 ± 0.59
Collegial nurse–physician relations	2.35 ± 0.61
**Nursing organisational culture**	
Relation‐oriented culture	3.07 ± 0.80
Innovation‐oriented culture	2.79 ± 0.80
Hierarchy‐oriented culture	3.67 ± 0.63
Task‐oriented culture	2.92 ± 0.64

Abbreviation: SD, standard deviation.

### 5.2 Frequency of Nurses' Work Interruptions During the MAP

Overall, the frequency of human‐initiated work interruptions (2.69 ± 1.28 points) was higher than that of environment‐initiated work interruptions (1.86 ± 0.79 points).

**FIGURE 1:**
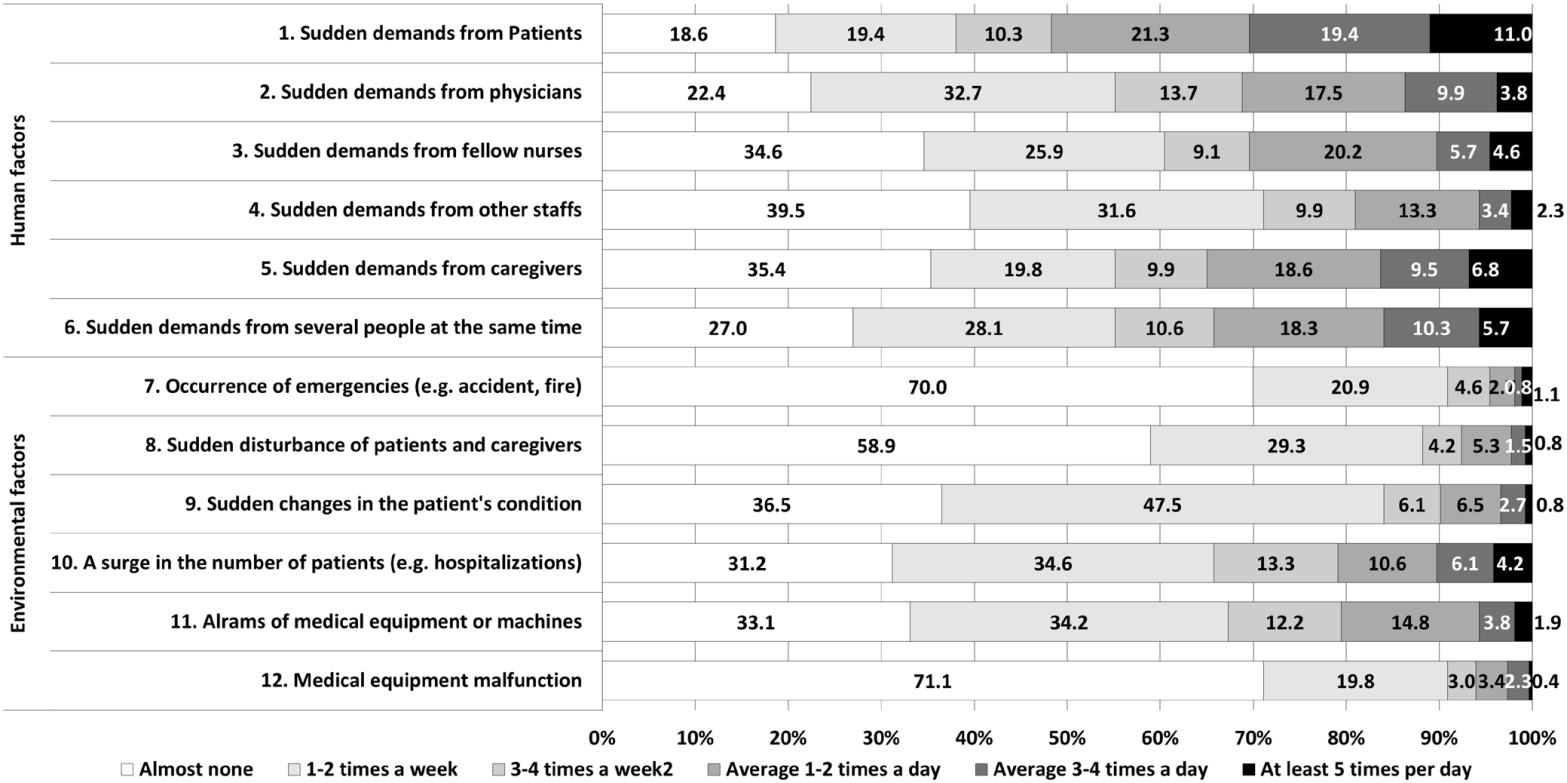
Response distribution for the frequency of nurses' work interruptions during the medication administration process.

Regarding responses indicating an average of 3–4 or more work interruptions per day (five points or more), among human‐initiated sources, interruptions caused by sudden demands from patients were the most frequent (30.4%), followed by caregivers (16.3%), several people at the same time (16.0%), physicians (13.7%), fellow nurses (10.3%) and other staff (5.7%). Among environment‐initiated sources, work interruptions caused by ‘a surge in the number of patients’ was the most common (10.3%), followed by ‘alarms of medical equipment or machines’ (5.7%), ‘sudden changes in the patient's condition’ (3.5%), ‘medical equipment malfunction’ (2.7%), ‘sudden disturbance of patients and caregiver’ (2.3%) and ‘occurrence of emergencies’ (1.9%). Figure 1 shows the response distribution for the frequency of nurses' work interruptions.

### 5.3 Impact of Nursing Work Environment and Organisational Culture on Nurses' Work Interruptions During the MAP

Analysis of the difference in frequency of work interruptions according to general characteristics revealed that there was a statistically significant difference according to ‘average number of patients per nurse per shift’ (*t* = −3.84, *p* < 0.001) for human‐initiated interruptions (Table 1).

In multiple linear regression analysis, the factors associated with human‐initiated interruptions were ‘average number of patients per nurse per shift’ (*β* = 0.17, *p* = 0.02), ‘staffing and resource adequacy’ (*β* = −0.16, *p* = 0.04), ‘relation‐oriented culture’ (*β* = 0.19, *p* = 0.03), and ‘innovation‐oriented culture’ (*β* = −0.22, *p* = 0.03). The factors associated with environment‐initiated interruptions were ‘female’ (*β* = −0.13, *p* = 0.04) and ‘task‐oriented culture’ (*β* = 0.17, *p* = 0.01) (Table 3).

**TABLE 3:** Factors related to nurses' work interruptions during the MAP (*N* = 263).

Variables	Human‐initiated					Environment‐initiated				
	*B*	SE	*β*	95% CI for *B* (lower, upper)	*p*	*B*	SE	*β*	95% CI for *B* (lower, upper)	*p*
Gender (ref. = male)										
Female	−0.50	2.26	−0.01	(−4.95, 3.94)	0.82	−2.91	1.44	−0.13	(−5.42, −0.08)	**0.04**
Age, years	0.14	0.19	0.09	(−0.22, 0.51)	0.45	−0.02	0.12	−0.02	(−0.25, 0.22)	0.89
Work experience as a nurse (ref. = ≤ 5 year)										
5–10 years	0.23	1.29	0.01	(−2.31, 2.76)	0.86	1.14	0.82	0.12	(−0.47, 2.76)	0.17
> 10 years	−1.51	2.37	−0.09	(−6.17, 3.16)	0.53	1.53	1.51	0.14	(−1.44, 4.51)	0.312
Type of ward (ref. = general ward)										
Intensive care unit	0.13	1.54	0.01	(−2.91, 3.17)	0.93	1.86	0.98	0.15	(−0.08, 3.80)	0.06
Emergency room	−2.74	2.49	−0.07	(−7.63, 2.15)	0.27	−1.99	1.58	−0.08	(−5.11, 1.13)	0.21
Average number of patients per nurse per shift	0.31	0.13	0.17	(0.04, 0.57)	**0.02**	0.13	0.09	0.11	(−0.04, 0.29)	0.14
Nursing work environments										
Nursing foundations for quality of care	0.12	0.16	0.06	(−0.19, 0.44)	0.45	−0.11	0.10	−0.09	(−0.31, 0.09)	0.28
Nursing managers' ability, leadership and support of nurses	−0.28	0.26	−0.09	(−0.79, 0.23)	0.28	0.06	0.17	0.03	(−0.26, 0.39)	0.70
Staffing and resource adequacy	−0.51	0.25	−0.16	(−1.00, −0.02)	**0.04**	−0.25	0.16	−0.12	(−0.57, 0.06)	0.11
Collegial nurse–physician relations	−0.29	0.32	−0.07	(−0.91, 0.33)	0.35	0.06	0.20	0.02	(0.34, 0.45)	0.77
Nursing organisational culture										
Relation‐oriented culture	1.82	0.82	0.19	(0.20, 3.44)	**0.03**	0.01	0.53	0.00	(−1.03, 1.04)	0.99
Innovation‐oriented culture	−2.09	0.93	−0.22	(−3.90, −0.26)	**0.03**	−0.15	0.59	−0.03	(−1.32, 1.01)	0.79
Hierarchy‐oriented culture	0.43	0.80	0.04	(−1.15, 2.00)	0.59	−0.05	0.51	−0.01	(−1.05, 0.96)	0.93
Task‐oriented culture	1.26	0.78	0.11	(−0.29, 2.80)	0.11	1.28	0.50	0.17	(0.29, 2.26)	**0.01**
	*R* ^2^ = 0.15, Adjust *R* ^2^ = 0.10, *F* = 2.91 (*p* < 0.001)					*R* ^2^ = 0.10, Adjust *R* ^2^ = 0.05, *F* = 1.83 (*p* = 0.03)				

*Note:* Bold values, *p* < 0.05.

Abbreviations: CI, confidence interval; MAP, medication administration process; SE, standard error.

## 6 Discussion

This study identified the frequency and associated factors of work interruptions during the MAP among nurses, classified by human‐ and environment‐initiated sources. The findings indicate that human‐initiated interruptions occurred more frequently than environment‐initiated ones. The key factors associated with these work interruptions included task‐related factors (average number of patients per nurse per shift) and organisational factors (staffing and resource adequacy, organisational culture), reflecting key components of the SEIPS model. These results demonstrate how multiple work system elements contribute to work interruption patterns during medication administration.

In this study, among human‐initiated sources, sudden demands from patients and caregivers were the most frequent. This finding may be explained by Korea's distinctive healthcare environment, particularly the continuous presence of family caregivers at bedsides and the emphasis on responding to patient demands and managing complaints (Son and Kim 2019; Park et al. 2022). However, patient‐ and caregiver‐initiated interruptions are not unique to the Korean context. Similar patterns have been documented in Western healthcare settings, where studies reported such interruptions during medication administration (Schroers 2018; Alteren et al. 2021), suggesting that while cultural factors may amplify their frequency in Korea, the underlying challenge is broadly shared. This cross‐contextual pattern points to common systemic issues, including inadequate communication systems between patients and nurses and insufficient pre‐admission education about hospital protocols that require attention across diverse healthcare settings. To mitigate such work interruptions, strategies are needed to systematically manage patient and caregiver needs (Wang et al. 2021). Educational tools such as bedside flyers that inform patients and caregivers about the importance of medication administration may help reduce sudden demands (Sims et al. 2024). Additionally, ‘Do not interrupt’ vests that clearly signal a nurse is performing medication rounds have been suggested, though evidence regarding their effectiveness remains inconsistent (Berdot et al. 2021).

Our study found that among environment‐initiated interruptions, a surge in patient volume was the most frequently reported source of interruptions. Additionally, human‐initiated interruptions increased as the average number of patients per nurse rose, while adequate staffing and resource availability were associated with fewer work interruptions. These findings suggest that maintaining an appropriate nurse‐to‐patient ratio and securing sufficient resources are essential for minimising work interruptions and promoting safety. In hospital settings, patient conditions are constantly changing, and frequent patient admissions, discharges, and transfers result in ongoing shifts in the actual number of patients each nurse must care for (Hopkinson and Wiegand 2017). These dynamic changes substantially increase nurses' workload, leading to significant strain that may interfere with critical tasks such as medication administration. These findings align with international studies that have identified workload as a source of interruptions (Alteren et al. 2021; Wang et al. 2024). However, the impact of workload‐related interruptions may be particularly pronounced in the Korean context, where nurse‐to‐patient ratios remain substantially higher than in both Western and other Asian healthcare systems (Cho et al. 2016). This disparity, combined with limited organisational flexibility in workforce allocation (Son and Kim 2019), leaves nurses with less buffer capacity to manage sudden patient surges, making them more vulnerable to environment‐initiated interruptions. This pattern illustrates how inadequate staffing amplifies interruption patterns, underscoring the universal importance of appropriate nurse‐to‐patient ratios and effective workload management as critical strategies to minimise interruptions and enhance medication safety across diverse healthcare contexts. To address these challenges, system‐level interventions are essential. Workflow redesign, role clarification, and deployment of additional staff during high‐demand periods can effectively prevent unnecessary interruptions and sustain safe medication practices (McGillis Hall et al. 2010). For instance, implementing a Medication Pass Time Out initiative, in which nurses are protected from non‐urgent interruptions during medication administration through the support of other staff who respond to patient calls and needs, can reduce the burden on nurses (Nguyen et al. 2010). Additionally, ensuring equitable distribution of staffing and resources can reduce nurses' workload burden and enhance medication workflow continuity (Liu et al. 2025). Such strategies are consistent with the systems approach, which emphasises environmental modification for error prevention rather than focusing on individual performance (Anderson and Webster 2001).

In line with the broader findings of the study by Hopkinson and Wiegand (2017), which described the medical nursing unit as a complex environment where cultural values, beliefs, and normative practices contribute to work interruptions, our study also found that nursing organisational culture was significantly associated with the occurrence of work interruptions. Specifically, relationship‐oriented, task‐oriented and innovation‐oriented cultures were each associated with different patterns of work interruptions. Nurses who perceived their organisational culture as more relationship‐oriented reported more frequent human‐initiated interruptions. Relationship‐oriented cultures emphasise interpersonal relationships among nurses, as well as mutual support and collaboration among team members, which in turn can foster teamwork based on trust and respect (Choi and Park 2019). However, in such cultures, communication for information exchange and brief social interactions are valued, which may lead to more frequent work interruptions (Hopkinson and Wiegand 2017). To address this, it is important to implement strategies such as education programs that raise awareness about work interruptions, helping nurses recognise and minimise them during critical tasks (McGillis Hall et al. 2010; Teigné et al. 2023). Additionally, in units that are strongly perceived as having a relationship‐oriented culture, designating a ‘Safe Zone’ or ‘No Interruption Zone’ where non‐urgent conversations are limited during the MAP may help nurses maintain focus (Yoder et al. 2015).

On the other hand, a task‐oriented culture was associated with work interruptions caused by environmental sources. A task‐oriented culture tends to emphasise stability, performance and productivity, which may lead nurses to pay closer attention to environmental changes (e.g., equipment alarms or patient status changes), resulting in more frequent work interruptions. Hopkinson and Wiegand (2017) similarly observed that nurses often felt responsible for managing all tasks themselves, which resulted in work interruptions. Previous research has also shown that nurses working in task‐oriented cultures are more likely to report medication errors (Lee and Lee 2021), suggesting increased sensitivity to safety‐related environmental cues. In such contexts, nurses often face dilemmas when deciding whether to continue their current task or respond to an interruption. Maintaining control and making sound decisions during these situations is crucial. Therefore, education and training on managing work interruptions and real‐time prioritisation should be integrated into clinical practice (Schroers 2018; Wang et al. 2024).

In contrast, the perception of an organisational culture as innovation‐oriented was associated with a decrease in the frequency of human‐initiated interruptions. An innovation‐oriented culture emphasises openness to new approaches, flexibility and adaptation to dynamic clinical environments (Choi and Park 2019; Han 2002). Such cultures may reduce unnecessary work interruptions and promote systematic task execution. For example, nurse‐led initiatives to manage work interruptions may reflect innovation‐oriented values in clinical settings (Sims et al. 2024). Therefore, fostering this type of culture may enhance nursing workflow efficiency and patient safety.

### 6.1 Strength and Limitations of the Work

This study offers valuable insights by specifically analysing the human and environmental sources associated with work interruptions in nurses' medication workflows. Notably, it highlights that sudden demands from patients and caregivers are significant contributors to these work interruptions and underscores the importance of appropriate staffing and resource allocation in ensuring smooth medication workflows and enhancing the efficiency of nursing care.

However, this study has several limitations. First, it was conducted exclusively with nurses working at tertiary hospitals. As the characteristics and frequency of work interruptions may vary depending on the context, future research should include different types of healthcare institutions to gain a broader understanding of the work interruptions frequency and associated factors. Second, as this study used convenience and snowball sampling methods and relied on a self‐reported online survey, the representativeness of the sample may have been limited and certain biases could have occurred (e.g., selection or recall bias). Third, the sample was predominantly female (95.4%), reflecting the characteristics of the recruited participants rather than the national nursing workforce. This gender imbalance may limit the generalizability of the findings. Fourth, as this study employed a cross‐sectional design, causal relationships between variables cannot be inferred, and this limitation should be considered when interpreting the results. Fifth, while this study adopted the SEIPS model as its theoretical framework, it did not fully capture the complex interactions among system components. Although this approach was valuable for identifying associated factors, it may not have adequately reflected the dynamic nature of work systems in real‐world clinical settings. Future research should consider using structural equation modelling to examine interaction effects or longitudinal designs to explore the evolving interplay among system components. Nevertheless, this study offers a comprehensive analysis of factors associated with work interruptions during the MAP and provides a strong foundation for developing strategies to reduce such interruptions.

### 6.2 Recommendations for Further Research

Although eliminating work interruptions during the MAP in healthcare settings is impossible, their impact must be studied thoroughly. Work interruptions not only increase the risk of medication errors, negatively affecting patient safety, but also contribute to nurse burnout and job dissatisfaction. Therefore, further research should employ various measurement methods to assess the prevalence and characteristics of work interruptions, while also considering nurses' perceptions and emotional responses to these interruptions.

Additionally, future studies should explore and evaluate system‐level interventions grounded in the systems approach. Longitudinal and interventional research is particularly needed to examine the effectiveness of strategies such as workflow redesign, staffing model optimization, and the development of organisational cultures that minimise unnecessary interruptions. Such research should assess the long‐term impact of these strategies on nursing efficiency and patient safety, thereby providing evidence for best practices that can be sustainably integrated into clinical settings.

### 6.3 Implications for Policy and Practice

The findings of this study have significant implications for healthcare policy and practice. To reduce work interruptions, healthcare institutions should implement practical strategies based on the characteristics and associated factors of interruptions. For example, educating patients and caregivers on the risks associated with interrupting medication administration is crucial in human‐initiated interruptions caused by patients or caregivers. This can be achieved by distributing educational materials or providing instructional videos at the time of hospital admission. In addition, nurse staffing is critical in interruptions; therefore, establishing protected times during which other staff members support the nurse administering medications is essential. To create a work environment that enables focused medication practice, establishing designated zones that preserve nurses' concentration during medication administration is also necessary. Further, cultivating a culture of innovation that empowers nurses to take initiative in managing work interruptions is necessary for adapting to changing environments and promoting patient‐centered care. Policymakers should prioritise proper staffing and resource allocation to reduce work interruptions during the MAP, enhancing nursing efficiency and patient safety.

## 7 Conclusion

This study provides valuable insights into the current state and associated factors of interruptions in nurses' medication workflows, categorised by human‐ and environment‐initiated sources. The findings underscore the critical importance of adequate staffing, resource support and organisational culture in mitigating such interruptions. Based on these findings, targeted strategies should be developed to address the specific sources and contexts of interruptions. Healthcare institutions should use these findings to improve staffing allocation and redesign workflows to support nurses' concentration during medication administration and protect them from unnecessary interruptions. Moreover, establishing a healthy work environment and organisational culture, including appropriate nurse‐to‐patient ratios and optimal staff allocation, is crucial for supporting uninterrupted medication workflows.

## Conflicts of Interest

The authors declare no conflicts of interest.

## Supporting information


**Table S1:** Measurement used in this study.

## Data Availability

The data that support the findings of this study are available from the corresponding author upon reasonable request.

## References

[jan70321-bib-0001] Alteren, J. , M. Hermstad , L. Nerdal , and S. Jordan . 2021. “Working in a Minefield; Nurses' Strategies for Handling Medicine Administration Interruptions in Hospitals—A Qualitative Interview Study.” BMC Health Services Research 21, no. 1: 1094. 10.1186/s12913-021-07122-8. 34649559 PMC8518177

[jan70321-bib-0002] Anderson, D. J. , and C. S. Webster . 2001. “A Systems Approach to the Reduction of Medication Error on the Hospital Ward.” Journal of Advanced Nursing 35, no. 1: 34–41. 10.1046/j.1365-2648.2001.01820.x. 11442680

[jan70321-bib-0003] Berdot, S. , A. Vilfaillot , Y. Bezie , et al. 2021. “Effectiveness of a ‘Do Not Interrupt’ Vest Intervention to Reduce Medication Errors During Medication Administration: a Multicenter Cluster Randomized Controlled Trial.” BMC Nursing 20, no. 1: 153. 10.1186/s12912-021-00671-7. 34429095 PMC8383384

[jan70321-bib-0004] Biron, A. D. , C. G. Loiselle , and M. Lavoie‐Tremblay . 2009. “Work Interruptions and Their Contribution to Medication Administration Errors: An Evidence Review.” Worldviews on Evidence‐Based Nursing 6, no. 2: 70–86. 10.1111/j.1741-6787.2009.00151.x. 19413581

[jan70321-bib-0005] Bower, R. , C. Jackson , and J. C. Manning . 2015. “Interruptions and Medication Administration in Critical Care.” Nursing in Critical Care 20, no. 4: 183–195. 10.1111/nicc.12185. 26084432

[jan70321-bib-0006] Cho, E. , M. Choi , E. Y. Kim , I. Y. Yoo , and N. J. Lee . 2011. “Construct Validity and Reliability of the Korean Version of the Practice Environment Scale of Nursing Work Index for Korean Nurses.” Journal of Korean Academy of Nursing 41, no. 3: 325–332. 21804341 10.4040/jkan.2011.41.3.325

[jan70321-bib-0007] Cho, E. , N.‐J. Lee , E.‐Y. Kim , et al. 2016. “Nurse Staffing Level and Overtime Associated With Patient Safety, Quality of Care, and Care Left Undone in Hospitals: A Cross‐Sectional Study.” International Journal of Nursing Studies 60: 263–271. 10.1016/j.ijnurstu.2016.05.009. 27297386

[jan70321-bib-0008] Choi, J. , and M. Park . 2019. “Effects of Nursing Organisational Culture on Face‐To‐Face Bullying and Cyberbullying in the Workplace.” Journal of Clinical Nursing 28, no. 13–14: 2577–2588. 10.1111/jocn.14843. 30811682

[jan70321-bib-0009] Danesh, V. , F. Sasangohar , A. S. Kallberg , E. B. Kean , J. J. Brixey , and K. D. Johnson . 2022. “Systematic Review of Interruptions in the Emergency Department Work Environment.” International Emergency Nursing 63: 101175. 10.1016/j.ienj.2022.101175. 35843150

[jan70321-bib-0010] Eid, T. , S. Machudo , and R. Eid . 2022. “Interruptions During Medication Work in a Saudi Arabian Hospital: An Observational and Interview Study of Nurses.” Journal of Nursing Scholarship 54, no. 5: 639–647. 10.1111/jnu.12765. 35064618

[jan70321-bib-0011] Faul, F. , E. Erdfelder , A. G. Lang , and A. Buchner . 2007. “G* Power 3: A Flexible Statistical Power Analysis Program for the Social, Behavioral, and Biomedical Sciences.” Behavior Research Methods 39, no. 2: 175–191. 17695343 10.3758/bf03193146

[jan70321-bib-0012] Han, S. J. 2002. “A Study on the Relationship Between Nursing Organizational Culture and Organizational Performance.” Journal of Korean Academy of Nursing Administration 8, no. 3: 441–456.

[jan70321-bib-0013] Holden, R. J. , P. Carayon , A. P. Gurses , et al. 2013. “SEIPS 2.0: A Human Factors Framework for Studying and Improving the Work of Healthcare Professionals and Patients.” Ergonomics 56, no. 11: 1669–1686. 10.1080/00140139.2013.838643. 24088063 PMC3835697

[jan70321-bib-0014] Hong, K. J. , H. Chung , and Y. M. Jo . 2023. “Relationships Between Alternative Nurse Staffing Level Measurements and Nurses' Perceptions of Nurse Staffing Level Adequacy, Fatigue, and Care Quality.” Journal of Nursing Management 2023: 1–12. 10.1155/2023/6060536. PMC11918521 40225666

[jan70321-bib-0015] Hopkinson, S. G. , and D. L. Wiegand . 2017. “The Culture Contributing to Interruptions in the Nursing Work Environment: An Ethnography.” Journal of Clinical Nursing 26, no. 23–24: 5093–5102. 10.1111/jocn.14052. 28833728

[jan70321-bib-0016] Jalali, A. , K. Babaei , A. Sharifi , et al. 2025. “Psychometric Properties of the Persian Version of Nursing Work Interruption Scale.” BMC Nursing 24, no. 1: 191. 10.1186/s12912-025-02837-z. 39972284 PMC11837290

[jan70321-bib-0017] Johnson, M. , P. Sanchez , R. Langdon , et al. 2017. “The Impact of Interruptions on Medication Errors in Hospitals: an Observational Study of Nurses.” Journal of Nursing Management 25, no. 7: 498–507. 10.1111/jonm.12486. 28544351

[jan70321-bib-0018] Kellogg, K. M. , J. S. Puthumana , A. Fong , K. T. Adams , and R. M. Ratwani . 2021. “Understanding the Types and Effects of Clinical Interruptions and Distractions Recorded in a Multihospital Patient Safety Reporting System.” Journal of Patient Safety 17, no. 8: e1394–e1400. 10.1097/pts.0000000000000513. 29994817

[jan70321-bib-0019] Kim, M. , and S. Oh . 2016. “Assimilating to Hierarchical Culture: A Grounded Theory Study on Communication Among Clinical Nurses.” PLoS One 11, no. 6: e0156305. 10.1371/journal.pone.0156305. 27253389 PMC4890802

[jan70321-bib-0020] Kim, M. S. , J. H. Kim , and S. J. Han . 2004. “The Development of the Nursing Organization Culture Measurement Tool.” Journal of Korean Academy of Nursing Administration 10, no. 2: 175–184.

[jan70321-bib-0021] Kim, Y. , M. J. Lee , M. Choi , E. Cho , and G. W. Ryu . 2023. “Exploring Nurses' Multitasking in Clinical Settings Using a Multimethod Study.” Scientific Reports 13, no. 1: 5704. 10.1038/s41598-023-32350-9. 37029189 PMC10082008

[jan70321-bib-0022] Kwon, Y. E. , M. Kim , and S. Choi . 2021. “Degree of Interruptions Experienced by Emergency Department Nurses and Interruption Related Factors.” International Emergency Nursing 58: 101036. 10.1016/j.ienj.2021.101036. 34332454

[jan70321-bib-0023] Lake, E. T. 2002. “Development of the Practice Environment Scale of the Nursing Work Index.” Research in Nursing & Health 25, no. 3: 176–188. 12015780 10.1002/nur.10032

[jan70321-bib-0024] Laustsen, S. , and L. Brahe . 2018. “Coping With Interruptions in Clinical Nursing—A Qualitative Study.” Journal of Clinical Nursing 27, no. 7–8: 1497–1506. 10.1111/jocn.14288. 29396916

[jan70321-bib-0025] Lee, H. Y. , and E.‐K. Lee . 2021. “Safety Climate, Nursing Organizational Culture and the Intention to Report Medication Errors: A Cross‐Sectional Study of Hospital Nurses.” Nursing Practice Today 20: 6704. 10.18502/npt.v8i4.6704.

[jan70321-bib-0026] Lin, T. , X. Feng , Y. Gao , et al. 2021. “Nursing Interruptions in Emergency Room in China: An Observational Study.” Journal of Nursing Management 29, no. 7: 2189–2198. 10.1111/jonm.13372. 33993569

[jan70321-bib-0027] Liu, Q. , Y. Lu , S. Zhai , C. Dai , C. Kan , and C. Chen . 2025. “Workflow Interruptions, Perceived Workload and Missed Nursing: Their Impact on Nurses' Health Status—A Structural Equation Model.” Journal of Advanced Nursing 58: 16855. 10.1111/jan.16855. 39991959

[jan70321-bib-0028] McGillis Hall, L. , C. Pedersen , and L. Fairley . 2010. “Losing the Moment.” Journal of Nursing Administration 40, no. 4: 169–176. 10.1097/nna.0b013e3181d41162. 20305462

[jan70321-bib-0029] Nguyen, E. E. , P. M. Connolly , and V. Wong . 2010. “Medication Safety Initiative in Reducing Medication Errors.” Journal of Nursing Care Quality 25, no. 3: 224–230. 10.1097/ncq.0b013e3181ce3ae4. 20535847

[jan70321-bib-0030] Park, J. Y. , J. F. Pardosi , M. S. Islam , T. Respati , K. Chowdhury , and H. Seale . 2022. “What Does Family Involvement in Care Provision Look Like Across Hospital Settings in Bangladesh, Indonesia, and South Korea?” BMC Health Services Research 22, no. 1: 922. 10.1186/s12913-022-08278-7. 35841023 PMC9286761

[jan70321-bib-0031] Schroers, G. 2018. “Characteristics of Interruptions During Medication Administration: An Integrative Review of Direct Observational Studies.” Journal of Clinical Nursing 27, no. 19–20: 3462–3471. 10.1111/jocn.14587. 29945303

[jan70321-bib-0032] Sims, T. , P. Narayanan , A. Alex , and M. J. Bacchus . 2024. “Decreasing Nonemergent Nurse Interruptions During Peak Medication Administration Time Utilizing ‘the Golden Hour’.” Journal of Nursing Care Quality 39, no. 2: 99–101. 10.1097/ncq.0000000000000750. 37782912

[jan70321-bib-0033] Son, H. M. , and E. Kim . 2019. “Nurse's Experiences of Work Flow Interruptions in Clinical Setting.” Journal of the Korean Association for Qualitative Research 4: 11–21. 10.48000/KAQRKR.2019.5.11.

[jan70321-bib-0034] Teigné, D. , L. Cazet , G. Birgand , et al. 2023. “Improving Care Safety by Characterizing Task Interruptions During Interactions Between Healthcare Professionals: An Observational Study.” International Journal for Quality in Health Care 35, no. 3: 69. 10.1093/intqhc/mzad069. PMC10507660 37688401

[jan70321-bib-0035] Trus, M. , N. Galdikiene , S. Balciunas , P. Green , M. Helminen , and T. Suominen . 2019. “Connection Between Organizational Culture and Climate and Empowerment: The Perspective of Nurse Managers.” Nursing and Health Sciences 21, no. 1: 54–62. 10.1111/nhs.12549. 30091283

[jan70321-bib-0036] Wang, Q. , X. Ding , M. Zhu , et al. 2024. “Experiences of Clinical Nurses With Medication Interruption: A Systematic Review and Qualitative Meta‐Synthesis.” Worldviews on Evidence‐Based Nursing 21, no. 6: 598–610. 10.1111/wvn.12749. 39392417

[jan70321-bib-0037] Wang, W. , L. Jin , X. Zhao , Z. Li , and W. Han . 2021. “Current Status and Influencing Factors of Nursing Interruption Events.” American Journal of Managed Care 27, no. 6: e188. 10.37765/ajmc.2021.88667. 34156222

[jan70321-bib-0038] Webster, C. S. 2022. “Existing Knowledge of Medication Error Must Be Better Translated Into Improved Patient Safety.” Frontiers in Medicine 9: 870587. 10.3389/fmed.2022.870587. 35655855 PMC9152084

[jan70321-bib-0039] Yoder, M. , D. Schadewald , and K. Dietrich . 2015. “The Effect of a Safe Zone on Nurse Interruptions, Distractions, and Medication Administration Errors.” Journal of Infusion Nursing 38, no. 2: 140–151. 10.1097/nan.0000000000000095. 25723837

[jan70321-bib-0040] Yu, E. J. , and E. N. Lee . 2022. “Development and Validation of a Nursing Work Interruption Scale.” International Journal of Environmental Research and Public Health 19, no. 20: 13487. 10.3390/ijerph192013487. 36294067 PMC9602459

[jan70321-bib-0041] Yu, E. J. , E. N. Lee , J. M. Kim , and H. J. Jun . 2019. “Concept Analysis of the Work Interruption by Nurses.” Journal of Korean Academy of Nursing Administration 25, no. 4: 272–281. 10.11111/jkana.2019.25.4.272.

