# Supplementary material for: Frequency and Associated Factors of Interruptions During the Medication Administration Process Among Nurses in South Korea: A Cross‐Sectional Study
Source: J Adv Nurs. 2025 Oct 28;82(6):6274–83. doi: 10.1111/jan.70321 (PMC13176717; doi:10.1111/jan.70321)
Supplement: Supplementary file 1 — Table S1: Measurement used in this study. [file JAN-82-6274-s001.docx]

Supplementary Table S1. Measurement used in this study

| Instrument | Domain (No. of items) | Response scale |
| --- | --- | --- |
| Nursing Work Interruption Scale  (Yu & Lee, 2022) | Human-initiated (6)  Environment-initiated (6) | 6-point Likert (1 = Almost none, 6= At least 5 times per day) |
| Korean version of the Practice Environment Scale of Nursing Work Index (K-PES-NWI)  (Cho et al., 2011) | Nurse participation in hospital affairs (9, excluded in this study)  Nursing foundations for quality of care (9)  Nurse managers’ ability, leadership, and support of nurses (4)  Staffing and resource adequacy (4)  Collegial nurse–physician relations (3) | 4-point Likert (1 = Strongly disagree, 4 = Strongly agree) |
| Nursing Organizational Culture Measurement Tool  (Kim et al., 2004) | Relation-oriented culture (5)  Innovation-oriented culture (6)  Hierarchy-oriented culture (5)  Task-oriented culture (4) | 5-point Likert (1 = Strongly disagree, 5 = Strongly agree) |
